# Perceptions of the Built Environment in Relation to Physical Activity and Weight Status in British Adolescents from Central England

**DOI:** 10.5402/2012/903846

**Published:** 2012-11-12

**Authors:** Michael J. Duncan, Samantha Birch, Lorayne Woodfield, Yahya Al-Nakeeb

**Affiliations:** ^1^Department of Biomolecular and Sports Sciences, Coventry University, Priory Street, Coventry CV1 5FB, UK; ^2^Department of Physical Education and Sports Studies, Newman University College, Birmingham B32 3NT, UK

## Abstract

The built environment may influence physical activity (PA) behaviour in young people. However, there is a dearth of data examining this issue in young people which considers weight status, physical activity, and environmental perceptions in the same analysis. Four hundred and five Year 10 pupils (223 boys, 182 girls, mean age ± S.D. = 14.8 ± 0.6 years), from central England, completed self-report measures of PA and perceptions of the built environment. Additionally, body mass index (BMI) was determined from height and weight. PA (MET/Min week^−1^) was positively related to environmental perceptions (*P* = 0.0001) and negatively related to BMI (*P* = 0.0001). PA was significantly greater in boys (*P* = 0.025) and normal weight children compared to girls and overweight/obese children, respectively (*P* = 0.01). Perception of the built environment was significant as a covariate (*P* = 0.0001) with a one-unit increase on this measure associated with a 141 MET/Min week^−1^ increase in PA. This study, therefore, supports claims that the built environment, and perceptions of it, can have an impact on health indices.

## 1. Background

The link between physical activity (PA) and health outcomes in children and adolescents is well established [1]. Low levels of PA are related to increased incidence of cardiovascular disease and obesity [1], and there have been calls for researchers to examine determinants of PA in children from differing backgrounds to accurately target health-enhancing interventions [2]. This is particularly important as children and adolescents are at increased health risk due to low levels of PA [3, 4].

PA behaviour can be seen as an interaction between personal attributes and environmental (built or social) factors [5] with these relating to predisposing, reinforcing and enabling factors for PA, respectively [6]. However, the majority of prior research has focused on personal and social environmental factors with little data on the physical/built environment [7]. Despite studies highlighting links between the physical environment and adult PA [8, 9], there is little data on this topic with younger populations [10] and studies using standardised measurement tools which are needed to more accurately inform interventions [11]. This is particularly the case for Europe where the built environment is very different from the US or Australia where most prior studies have been conducted [11]. Moreover, few studies have examined the relation between PA and perceptions of the environment in adolescents particularly drawing from a sample spread across socioeconomic status groups (SES) within one city. To date, only one study has examined this issue in Portuguese girls, reporting that higher SES was associated with higher PA and more positive perceptions of the environment than their lower SES counterparts. The aim of this study was to examine the relation between PA and perceptions of the built environment in a sample of low SES adolescents from a deprived area of central England. A second objective of the study was to examine gender and weight status differences in PA whilst accounting for environmental perceptions.

## 2. Methods

### 2.1. Participants

Year 10 pupils (*n* = 405, 223 boys, 182 girls, 80.2% Caucasian, mean age ± S.D. = 14.8 ± 0.6 years) volunteered to participate following institutional ethics approval, parental informed consent, and child assent from two secondary schools in central England. The children were drawn from four electoral wards within the city of Coventry with one school selected from each quartile of wards based on deprivation [12]. 

### 2.2. Procedures

Physical activity was assessed using the International Physical Activity Questionnaire [13] long form, as this has previously been used to assess habitual physical activity in children of the ages in the present study [14]. Details regarding the IPAQ have been extensively reported previously, and administration of this form was conducted according to recommended protocols [14, 15]. However, in the current study data are presented as (MET/Min week^−1^) in line with recommendations for scoring of the IPAQ measure [13]. The IPAQ questionnaire is scored to provide a measure of Metabolic Equivalents (METs) to yield a score in MET-minutes, whereby METs are defined as multiples of the resting metabolic rate and a MET-minute is computed by multiplying the MET score of an activity by the minutes it is performed for. MET-minute scores are equivalent to kilocalories for a 60-kilogram person. When presented as MET/Min week^−1^ this provides a measure of total weekly physical activity [13].

Perceptions of the built environment were assessed using the European environmental questionnaire ALPHA short form [16]. This questionnaire assesses perceptions of physical-activity-related environmental factors specific to the European population [17]. Participants respond to 11 questions on a 4-point Likert scale. The questions refer to key areas of the built environment including local facilities, neighbourhood aesthetics, traffic, crime, walking/cycling accessibility, and housing types. Scores range from 11 to 44 with higher scores reflecting more positive perceptions of the built environment.

Height (m) and mass (kg) were also assessed using a Seca stadiometre and weighing scales (Seca Instruments Ltd, Germany) from which body mass index (BMI kg/m^2^) was determined and participants' weight status classified using IOTF criteria [18].

### 2.3. Statistical Analysis

The relations between PA (MET/Min week^−1^), BMI, and perception of the built environment were assessed using Pearson's product moment correlations. A 2 (gender) by 2 (normal weight versus overweight/obese) ways Analysis of Covariance (ANCOVA) using environmental perceptions as a covariate was employed to examine differences in PA according to gender and weight status whilst accounting for any association between PA and environmental perceptions. Moreover, linear regression was employed to determine how much of the variance in both PA and BMI could be predicted by perception of the environment. PASW version 17 was used for all analyses. An alpha value of *P* = 0.05 was used to set statistical significance a priori.

## 3. Results

**Figure 1:**
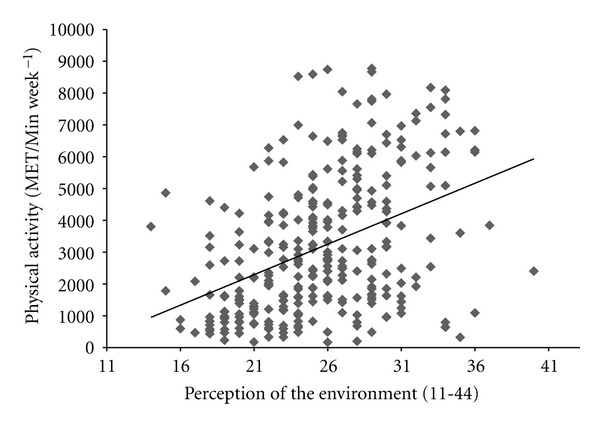
The relationship between physical activity (MET/Min week^−1^) and perception of the built environment.

**Figure 2:**
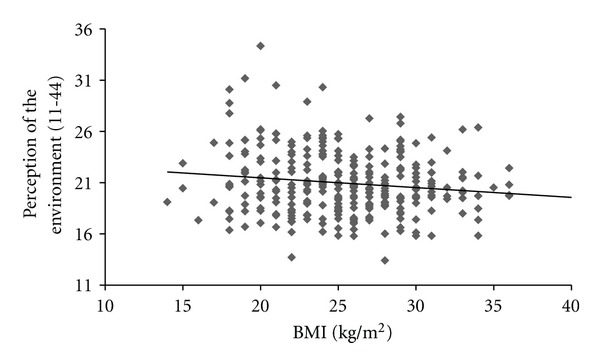
The relationship between BMI (kg/m^2^) and perception of the built environment.

ANCOVA indicated a significant gender difference in PA (*F*_1,401_ = 8.824, *P* = 0.025, partial *η* ^2^ = 0.22) with boys reporting greater PA than girls. Normal weight children were significantly more physically active than their overweight/obese peers (*F*_1,401_ = 6.775, *P* = 0.01, partial *η* ^2^ = 0.17). Mean ± S.D. of physical activity across gender and weight status groups is presented in Table 1. Furthermore, perception of the built environment was also significant as a covariate (*F*_1,401_ = 47.99, *P* = 0.0001, partial *η* ^2^ = 0.107). Slope parameter estimates (*β* = 141.2, *P* = 0.0001) revealed that a one-unit increase in perception of the built environment was associated with a 141.2 MET/Min week^−1^ increase in PA irrespective of gender and weight status. Results also indicated a significant, positive relationship between PA (MET/Min week^−1^) and perceptions of the environment (*r* = 0.367, *P* = 0.0001, see Figure 1) and BMI and perceptions of the environment (*r* = −0.214, *P* = 0.0001, see Figure 2) with more positive perceptions of the built environment being related to higher levels of PA and lower BMI, respectively. Linear regression identified a significant model for PA (*F*_1,401_ = 62.5, Adjusted *R* ^2^ = 0.132, *P* = 0.01) whereby perception of the environment predicted 13.2% of the variance in PA. There was also a significant model for perception of the environment predicting BMI in this population (*F*_1,401_ = 19.5, adjusted *R* ^2^ = 0.044, *P* = 0.01), with environmental perceptions predicting 4.4% of the variance in BMI.

## 4. Discussion

This study aimed to better understand the links between perceptions of the built environment and PA in adolescents in central England. These results support prior studies that have reported boys and normal weight adolescents to be more physically active than girls and overweight/obese adolescents, respectively [1]. The current study adds to the extant knowledge base in this area by presenting data on the association between perceptions of the built environment, PA, and weight status in adolescents. These results indicate that more positive perceptions of the built environment are associated with greater levels of habitual PA and healthy weight status. However, this study is limited due to its cross-sectional nature and in the case of the above association (see Figure 1) and the association presented in Figure 2 it cannot be deduced whether perception of the built environment is influenced by physical activity and/or BMI or whether physical activity and/or BMI is influenced by the perception of the environment. Further studies using a longitudinal or randomised controlled trial design would therefore be useful in answering this question. Despite this, to the authors' knowledge, this is the first study to evidence this association in an adolescent population. However, these findings do complement existing literature that suggests PA behaviour is enhanced when the built environment is more conducive to PA and where perceptions of the environment are more positive [19]. The current research also contradicts prior research with British children that reported no association between perceptions of the environment and overall PA [11]. This latter study employed a heterogeneous population of children drawn from across different SES groups and it may be that the association identified in this study is location or SES group specific. Further larger scale comparative studies between high and low SES groups would be needed to verify this claim. Moreover, in the current study, a validated self-report measure of perceptions of the built environment was employed whereby a total environmental perception score is obtained. Although this exploratory study provides some useful information, the authors recognise that this measure has limitations and the use of a total environmental perceptions score does not provide explicit indication as to what aspect of the built environment may be more important in influencing physical activity and weight status in young people. Moreover, given that only a small amount of the variation in BMI was predicted by the questionnaire measure in this study, it may be that objective measures of the built environment, rather than perception, would produce a stronger predictive model. As such further larger scale research using more comprehensive and qualitative measures of the built environment may be needed to build on the work presented here. In addition, as it is not clear yet whether the objectively assessed or perceived environment is more influential [7] in adolescents' PA behaviour, additional studies incorporating objective measures of PA and the built environment would be useful in developing understanding of this area. Despite this, the novel, exploratory data presented here supports the notion that PA in youth is associated with modifiable factors of the built environment [19]. This is important as it has implications for policy makers to prioritise development of environments that are conducive to enhancing PA (e.g., reducing crime rate, increased street connectivity, increased greenspace, and local facilities) in deprived areas of the United Kingdom.

## Figures and Tables

**Table 1:** Mean ± S.D. of physical activity in low SES adolescents from central England across gender and weight status groups.

	Physical activity (MET/Min week^−1^)		*P*
	Mean	S.D.
Boys (*n* = 223)	3088.4	1829.6	0.025
Girls (*n* = 182)	2526.4	2002.5	
Normal weight (*n* = 295)	3085.4	1914.9	0.01
Overweight/obese (*n* = 110)	2529.4	1799.6	
